# Reframing workplace safety, wellbeing, and performance among operating room nurses in contemporary healthcare systems

**DOI:** 10.3389/fpubh.2026.1900907

**Published:** 2026-08-26

**Authors:** Liying Zhang, Yun Feng, Dan Li, Dongfang Liu

**Affiliations:** Department of Anesthesia Operation, Zhejiang Hospital, Hangzhou, China

**Keywords:** nurse wellbeing, operating room nurses, patient safety, perioperative performance, workplace safety

## Abstract

Operating rooms are complex, high-risk, and technology-intensive environments in which nurses perform safety-critical roles. This review aimed to reframe workplace safety, wellbeing, and performance among operating room nurses as interconnected system-level determinants of perioperative care. Using a thematic narrative approach and conceptual scope, literature published mainly between 2020 and 2026 was reviewed, with selected foundational studies included where conceptually necessary. Evidence was synthesized across occupational hazards, psychosocial stress, emotional labor, burnout, compassion fatigue, quality of work life, teamwork, safety culture, distractions, cognitive load, ergonomics, robotic surgery, artificial intelligence, perioperative competency, empowerment, retention, and workforce sustainability. The findings indicate that unsafe or poorly supported operating room environments expose nurses to physical, psychological, cognitive, ergonomic, and technological pressures that may compromise wellbeing and professional performance. Nurse wellbeing supports attention, communication, emotional regulation, resilience, clinical judgment, and participation in safety practices. Conversely, burnout, compassion fatigue, interruptions, hierarchical communication, ergonomic strain, and inadequate technology readiness may weaken vigilance, teamwork, procedural reliability, and patient-safety margins. Operating room nursing performance is therefore multidimensional, encompassing technical competence, infection-prevention behavior, situational awareness, adaptability, speaking-up behavior, and technology-enabled workflow coordination. Treating nurse safety and wellbeing as patient-safety and system-performance priorities may strengthen surgical care, teamwork, retention, and the sustainability of perioperative healthcare systems.

## 1 Introduction

Operating rooms are among the most complex, high-risk, and technology-intensive environments in contemporary healthcare (1). Patient safety in this setting depends on technical precision, aseptic vigilance, timely decision-making, and effective interprofessional coordination. Operating room nurses contribute directly to these processes by maintaining the sterile field, preparing instruments and equipment, coordinating surgical workflow, advocating for patients, completing documentation, and monitoring intraoperative risks (2). They should therefore be recognized as safety-critical members of the perioperative team rather than as providers of procedural support alone (3, 4). With the growing complexity of surgery, patients' acuity, technological advances, and institutional demands for efficiency, the functions of operating room nurses have grown significantly. Contemporary operating rooms increasingly incorporate robotic platforms, advanced imaging, digital documentation, and intelligent technologies (5). Consequently, nursing performance now requires more than task completion. It depends on technical competence, situational awareness, clear communication, adaptability, emotional regulation, and readiness to use emerging technologies (6). The operating room nursing field is a space where the influence of human expertise and conditions at the system level is increasingly evident in recent studies on perioperative competency, intelligent technologies, robotic surgery, and dedicated perioperative teams (7).

Despite these increased responsibilities, the safety and wellbeing of operating room nurses are still frequently viewed as occupational health concerns rather than as integral components of clinical performance and patient safety. This restrictive perspective is increasingly outdated. In the operating room, nurses' performance cannot be separated from their working conditions (8). Physical hazards and psychosocial stress, cognitive overload, emotional exhaustion, ergonomic strain, interruptions, and weak safety culture can impair a nurse's capacity to pay attention, communicate concerns, foresee what is needed in a procedure, and respond to the risks that arise (9). Thus, the safety and health of operating room nurses should not be treated as an “issue on the periphery of the workforce” but as a crucial part of safe surgical systems (10).

Workplace safety in operating room nursing is multidimensional. Physical and occupational risks include sharps injuries, exposure to blood and body fluids, anesthetic gases, radiation, prolonged standing, musculoskeletal strain, and equipment-related hazards (11). Safety also depends on organizational and psychosocial conditions, including adequate staffing, clear roles, supportive leadership, non-punitive communication, and access to necessary resources (12). During surgery, these workplace conditions influence both nurses' wellbeing and their professional performance. Evidence concerning distractions and quality of work life among operating room nurses suggests that interruptions and environmental disruptions may impair attention, coordination, and work-related wellbeing (13). Similarly, research on compassion fatigue indicates that sustained emotional strain may compromise patient safety and workforce stability (14). The relationship between safety, wellbeing, and performance is also shaped by teamwork and communication. Operating room nurses work within highly interdependent teams, where leadership, role clarity, hierarchy, and psychological safety influence their ability to coordinate care and raise safety concerns (15). Rigid hierarchies, intimidation, or bullying may discourage nurses from speaking up or interrupting unsafe practices. Conversely, supportive teamwork and clear professional roles can strengthen vigilance, trust, and workflow efficiency (16).

Technological change introduces an additional layer of complexity. Artificial intelligence and precision-nursing technologies may support clinical decision-making, workflow monitoring, documentation, and perioperative safety (7). However, these technologies must be integrated carefully into nursing practice. Although robotic systems may reduce some physical demands, operating room nurses may continue to experience ergonomic strain and require specialized training (17). Technological advancement should therefore not be assumed to improve safety or performance unless it is accompanied by human-centered implementation, appropriate education, and organizational support.

This review synthesizes recent evidence to examine workplace safety, wellbeing, and performance among operating room nurses as interconnected determinants of perioperative care quality.

## 2 Thematic narrative approach and conceptual scope

This review utilizes a thematic narrative structure to present a synthesis of recent evidence pertaining to workplace safety, wellbeing and performance in operating room nurses. This is suitable as the literature is wide and the methodology varies, from integrative reviews and qualitative studies to cross-sectional studies, meta-syntheses, ergonomic analysis, competency-based investigations, workforce studies, and technology-focused reviews (18). Instead of statistically aggregating results, the review categorizes existing evidence conceptually to help describe the relationship between the OR nurse work environment and psychological wellbeing, work performance, patient-safety contribution, and workforce sustainability (19). Because this was a thematic narrative review rather than a systematic review, the identified literature was used to develop, contextualize, and illustrate the principal themes rather than to constitute an exhaustively enumerated sample of included studies.

The major academic databases and scholarly sources such as PubMed/MEDLINE, Scopus, Web of Science, CINAHL, Google Scholar, and leading journals in the fields of nursing, perioperative practice, occupational health, patient safety, and healthcare technology were searched for relevant literature. The review focused on studies published between 2020 and 2026, in particular 2025–2026 studies on the following topics: infection prevention, quality of work life, distractions, teamwork, compassion fatigue, ergonomics in robotic surgery, perioperative competency, nurse retention, and artificial intelligence in operating room nursing (20, 21). Search terms were organized into four conceptual groups. The first addressed the professional group and setting, using terms such as “operating room nurse” and “perioperative nurse.” The second addressed occupational outcomes, including workplace safety, wellbeing, burnout, compassion fatigue, and quality of work life. The third covered performance-related concepts such as teamwork, communication, competency, and patient safety. The final group addressed emerging work-system issues, including ergonomics, robotic surgery, retention, and artificial intelligence (22). The review is deliberately integrative in scope. In addition to the traditional occupational risks, e.g. exposure to infection, sharps injuries, anesthetic gases, radiation, and musculoskeletal strain, workplace safety is also explored in the context of organizational and psychosocial factors, e.g. workload, staffing, leadership, team culture, psychological safety, quality of communication, and availability of resources (23). Wellbeing is understood as a professional resource to help manage emotions, attention, resilience, job satisfaction and retention. Performance is defined as a multifaceted phenomenon that includes technical skill, practice of infection prevention measures, situational awareness, communication accuracy, teamwork, adaptability, readiness to think, and use of technology (24, 25).

The literature selected was categorized into the key thematic areas which are salient to the current issues in operating room nursing: workplace safety, psychosocial stress, emotional labor, burnout, compassion fatigue, teamwork, safety culture, distractions, cognitive load, ergonomics, robotic surgery, competency, technology readiness, and workforce sustainability. This thematic structure also enables the review to transcend a focus on individual risk factors. It considers workplace safety, wellbeing, and performance as interdependent system-level determinants of the quality of perioperative care (26). In this context, outcomes among operating room nurses are fundamental to surgical safety, workflow effectiveness, and the sustainability of contemporary healthcare systems.

## 3 Conceptual reframing of safety, wellbeing, and performance

The concerns of workplace safety, wellbeing, and performance for operating room nurses should not be thought of as independent of each other or as occurring in isolation, one after the other. These domains are all interdependent parts of a single clinical work system in modern perioperative practice (27). The operating room is a high-hazard, high-pressure, technologically complex interprofessional communication, staffing, and organizational culture context in which physical hazards and other factors combine to impact both the nurse's experience and the ability to perform safety-critical tasks (28). Thus, nurse safety extends beyond protection from occupational injury; it is a foundational condition that supports sustained vigilance, clear communication, procedural awareness, and intraoperative safety-related decision-making (29).

The first part of this conceptual reframing is the understanding that working environments have a direct impact on nurse wellbeing (30). Unsafe or poorly supported operating room environments expose nurses to several categories of risk. Physical and occupational risks include infection, sharps injuries, radiation, anesthetic gases, prolonged standing, and musculoskeletal strain. At the same time, interruptions, psychological stress, and inadequate organizational support may increase cognitive and emotional burden (12). Compassion fatigue has emerged as a growing concern because operating room nurses frequently encounter emotionally demanding, high-pressure situations that require precision, composure, and constant readiness (31). Likewise, studies on ergonomics in robotic and laparoscopic surgery suggest that while technology can adjust the physical demands of a surgery, it does not necessarily reduce the musculoskeletal load or the requirement for ergonomic preparation (32, 33).

The second aspect is the placement of wellbeing as a professional tool as opposed to an individual quality. Wellbeing in the operating room helps to facilitate thinking and relationships needed for safe performance (34). When nurses have adequate psychological and physical resources, they are more likely to maintain situational awareness, monitor aseptic practice, raise concerns, and respond effectively to unexpected intraoperative events (35). Fatigue, burnout, emotional labor, compassion fatigue and quality of work life, on the other hand, can affect attention, weaken communication, decrease motivation and compromise clinical judgment (36). This relationship can also be supported by studies on quality of work life and distractions among OR nurses, which reveal how work environment interruptions and conditions relate to the professional experience and working capacity of nurses (37).

The third aspect relates to professional performance as a multifaceted result. Operating room nursing performance should not be evaluated solely by task completion or speed. It includes technical competence, instrument readiness, infection-prevention behavior, accurate communication, team coordination, adaptability, emotional regulation, reliable documentation, and technology readiness (38). The findings in the evidence regarding competency in the perioperative environment indicate specialized training, years of experience, empowerment, resilience and self-efficacy as significant factors associated with the professional competency of operating room nurses (39). This means the level of performance is a result of the combined efforts of individual competence and enabling workplace conditions, and not individual competence alone.

The fourth component connects nurse performance with patient safety and organizational outcomes. Aseptic vigilance, coordination and anticipation of risk are characteristics of operating room nurses that play a role in surgical safety (7). Fatigue, stress, the lack of teamwork or an unsafe environment may impact not only the nurse's performance but also the efficiency of the workflow, the prevention of infection, the reliability of communication and the margins of patient safety (40). Evidence from teams also suggests that collaboration between operation room nurses and other practitioners is effective when there is role awareness, leadership, psychological safety, resources, and a culture of the organization. These are what can make or break nurses' ability to speak up, coordinate efficiently, and be fully involved in the prevention of harm (41).

**Figure 1:**
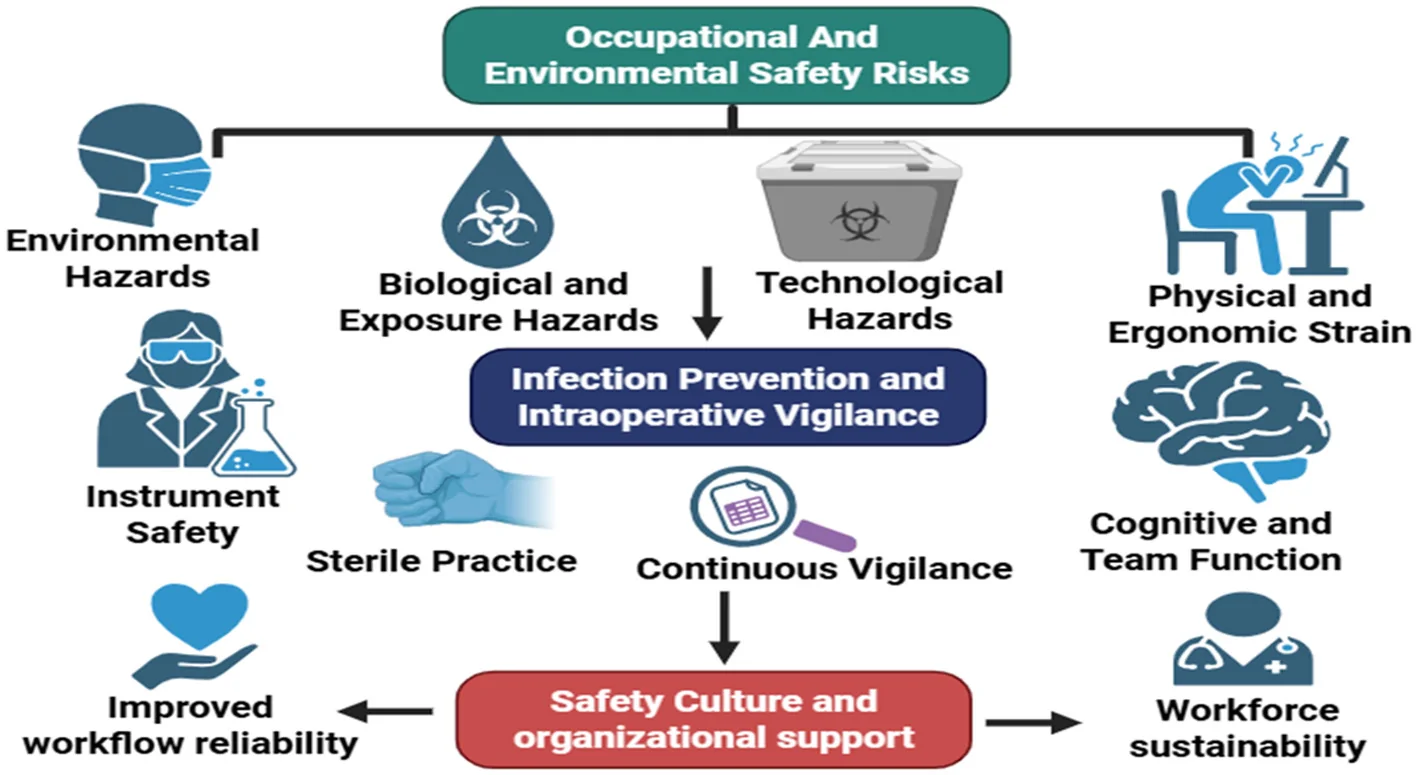
Conceptual framework illustrating how workplace safety conditions, psychosocial stressors, organizational culture, and technological factors influence operating room nurses' wellbeing and performance, ultimately affecting patient safety, surgical quality, workflow reliability, and workforce sustainability.

Taken together, these relationships form the conceptual framework presented in Figure 1. The framework positions workplace safety conditions, psychosocial stressors, organizational culture, and technological factors as upstream determinants of operating room nurses' wellbeing and professional performance, with downstream implications for patient safety, surgical quality, workflow reliability, and workforce sustainability. The major determinants linking workplace safety, wellbeing, professional performance, and patient safety are summarized in Table 1.

**Table 1:** Determinants of workplace safety, wellbeing, and performance among operating room nurses.

Determinant domain	Specific factors	Effect on wellbeing	Effect on performance	Patient-safety implication	References
Psychosocial determinants	Stress, emotional labor, fear of error, high accountability, uncertainty	Increases fatigue, anxiety, emotional exhaustion, and psychological strain	Reduces attention, confidence, communication clarity, and decision-making capacity	Greater risk of missed cues, delayed responses, and reduced vigilance	(98)
Organizational determinants	Staffing, workload, leadership, resources, reporting systems, safety climate	Shapes job satisfaction, perceived support, burnout risk, and retention intention	Influences guideline adherence, workflow reliability, and task completion	Determines whether safety practices are consistently supported or undermined	(40, 175)
Team-based determinants	Role clarity, team familiarity, trust, leadership, professional respect	Promotes belonging, confidence, and psychological support	Improves coordination, anticipation, communication, and shared situational awareness	Supports timely risk recognition and coordinated prevention of harm	(176)
Hierarchical and cultural determinants	Surgeon–nurse hierarchy, punitive culture, power distance, speaking-up barriers	Increases stress, silence, moral distress, and perceived lack of control	Weakens assertive communication and reduces participation in safety decisions	Safety concerns may remain unreported or unresolved	(177, 178)
Cognitive and environmental determinants	Noise, interruptions, multitasking, documentation burden, procedural complexity	Increases mental workload and frustration	Disrupts concentration, memory, sequencing, and communication accuracy	Increases vulnerability to communication failure and workflow disruption	(179, 180)
Ergonomic determinants	Prolonged standing, awkward posture, repetitive movements, equipment handling	Contributes to pain, physical fatigue, and musculoskeletal strain	Reduces stamina, comfort, and sustained procedural focus	Physical strain may compromise vigilance during long procedures	(181)
Technological determinants	Robotic systems, AI tools, digital documentation, equipment complexity	May reduce workload when well-designed but increase digital stress if poorly integrated	Supports efficiency and decision-making when usable; may increase cognitive load when fragmented	Technology can improve safety only when aligned with workflow and training	(148, 182)
Professional determinants	Training, experience, competency, empowerment, self-efficacy, mentorship	Strengthens confidence, resilience, engagement, and professional identity	Improves technical competence, adaptability, and safety participation	Enhances infection prevention, procedural reliability, and risk anticipation	(183)

## 4 Workplace safety as the foundation of perioperative performance

**Figure 2:**
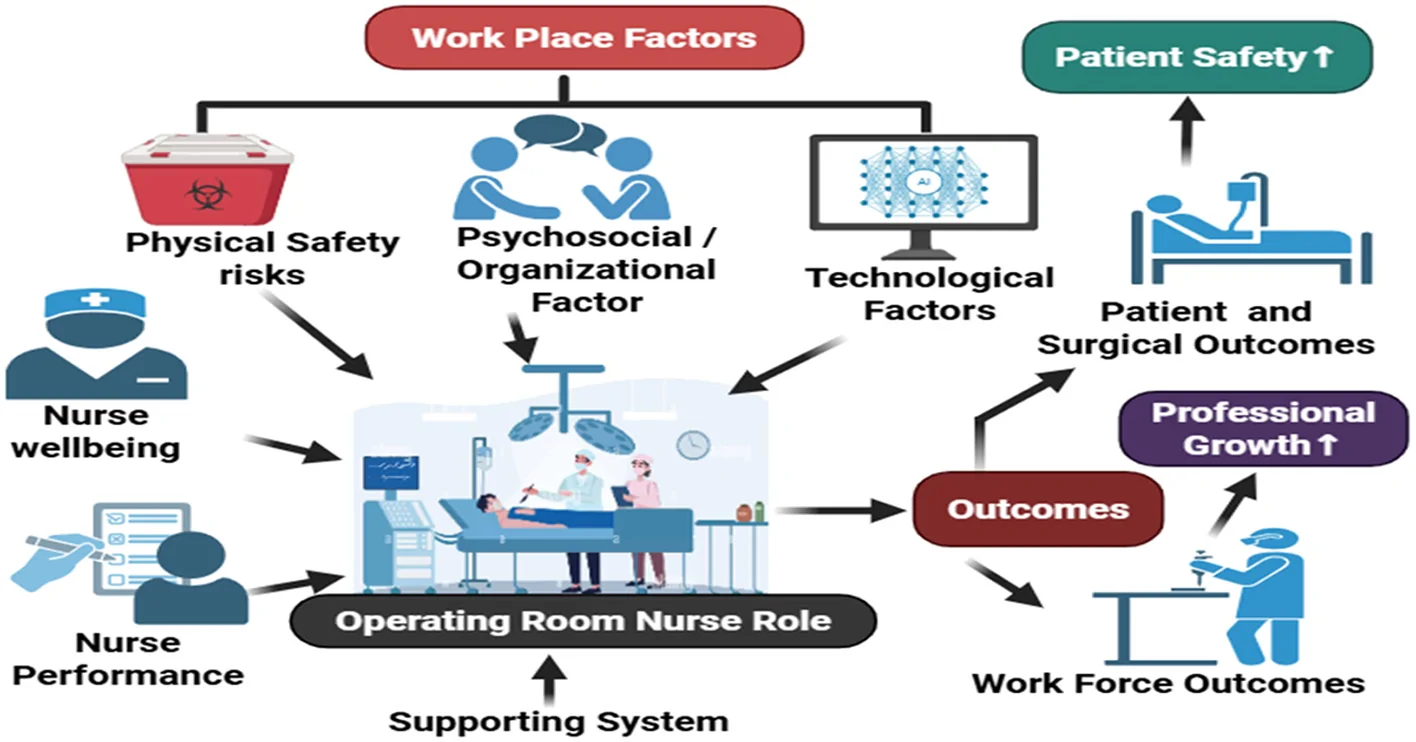
Workplace safety in operating room nursing involves occupational risk control, infection-prevention vigilance, ergonomic protection, and supportive organizational culture. Together, these factors strengthen perioperative performance, improve patient safety, and support workforce sustainability.

The operating room places substantial physical, cognitive, and organizational demands on nurses, making workplace safety fundamental to high-quality performance (42). In the operating room, the meaning of safety is not only to minimize injury to the nurse, but also to define the ability of the nurse to use aseptic technique, anticipate the needs of the surgeon, communicate risks, facilitate the surgical process and prevent injury to the patient (43). A safe perioperative work environment is therefore both a prerequisite for reliable performance and an occupational health requirement (Figure 2) (44).

### 4.1 Occupational and environmental safety risks

Operating room nurses experience interacting biological, chemical, physical, ergonomic, and technological hazards (45). Biological risks include sharps injuries and exposure to blood, body fluids, and infectious material. Chemical and physical risks include anesthetic gases, radiation, noise, and equipment-related hazards. Prolonged standing, awkward postures, fatigue, and the handling of heavy equipment add an important ergonomic dimension (12).

Sharps injuries and contact with blood or body fluids are still major safety concerns as Operating Room Nurses are constantly working with surgical instruments, needles, scalpel, contaminated tissues, etc. during the operation (46). The risks are exacerbated by time pressure, spatial constraints, unforeseen procedural changes and fast instrument exchange. Besides, radiation exposure is growing to be more important in image-based and technology-supported interventions (47). The studies have highlighted the ongoing need for radiation awareness and protective practices among operating room staff, as well as the importance of staff education in the context of routine use of imaging technologies (48). Likewise, the work environment of health professionals in the perioperative fields is also an important environmental issue, due to the presence of anesthetic gases, for which there is a need for monitoring systems, ventilation controls, and institutional safety procedures (49).

Another key aspect of workplace safety is physical strain. Operating room nurses are frequently required to stand for extended periods, have to assume awkward postures, repeat certain tasks, lift or position equipment and work in an environment where space is limited (50). These physical stresses can lead to fatigue, musculoskeletal discomfort, reduced stamina and decreased concentration during lengthy procedures (51). These risks are particularly relevant when surgeries are lengthy or when there is a shortage of personnel so that staff members cannot rotate or recover. Thus, occupational safety in the operating room is a multifaceted problem that encompasses exposure control, ergonomic protections, fatigue prevention and workflow design (11).

### 4.2 Infection prevention and intraoperative vigilance

One of the most obvious and safety-related jobs of operating room nurses is infection prevention (52). They are responsible for maintaining the sterile field, preparing and managing sterile instruments, and detecting breaks in sterility, as well as providing assistance with proper surgical attire and environment controls and ensuring adherence to infection-prevention guidelines (53). These are actions that must be continually monitored, as the risk of infection during surgery depends not only on the technical aspects, but also on the behavior of the team, availability of resources, communication and organizational culture (54).

The intraoperative phase's infection prevention relies on many conditions, such as the staff's professional competence, evidence-based infection prevention recommendations, teamwork, resources, and conditions that facilitate safe working (55). This is significant to the current review as it indicates that infection prevention is not exclusively the responsibility of one nurse (56). Instead, infection prevention is a system-level performance outcome that depends on nurses having the necessary knowledge, authority, time, equipment, and team support. Even very capable nurses can struggle to keep infection-prevention standards optimal under these circumstances (57).

Intraoperative vigilance is also associated with workplace safety and performance (58). The surgical floor nurse is required to constantly observe the instruments, sterility, the sequence of the surgical procedure and the risks to the environment and be able to predict what the surgical team will need (59). Fatigue, distractions, equipment failures, poor communication, or insufficient workforce can all be factors that affect this vigilance. The care and cleaning of surgical instruments further exemplify how nursing vigilance plays a crucial role in preventing contamination and ensuring the proper readiness of surgical instruments (60). In this context, infection prevention should be viewed as a performance process, and not a checklist activity. It requires focused attention, procedural control, cross-disciplinary collaboration, and the skills to identify and resolve safety issues in a timely manner before they escalate into a patient safety issue (61).

### 4.3 Safety culture and organizational support

Organizational culture plays an important role in the safety of the workplace in the operating room (62). Although policies and procedures are essential, their effectiveness also depends on adequate staffing, leadership support, interprofessional collaboration, available resources, reporting systems, and psychological safety (63). A well-established safety culture promotes early detection of risk, non-punitive reporting, respectful communication, and joint responsibility for patient safety (64). Conversely, poor safety culture can lead to a culture of unsafe shortcuts, a culture of not reporting, and an environment where hazards are not acted upon (65).

Organizational support is especially vital given that operating room nurses work in a complex interprofessional system (31). If nurses are understaffed, under-equipped, lacking in communication or adequate managerial support, then it is impossible to maintain safety through individual vigilance (66). Teamwork evidence indicates that team members' interdependence between operating room nurses and other practitioners is related to awareness of roles, leadership, resources, professional respect, and psychological safety (67). These factors contribute to whether or not nurses are able to convey risks, interrupt unsafe habits, and fully engage in intraoperative decision-making (41, 68).

Another key component of safety learning is a non-punitive environment. Near misses, equipment issues, breaches of sterility and communication may not be reported when nurses are afraid of being blamed or of a conflict situation (69). By contrast, supportive leadership and effective reporting structures allow safety events to be used for system improvement (70). In addition, information-technology-supported management strategies can make a difference in the operating room service quality and satisfaction of staff when they are part of a larger quality improvement initiative (71). Organizational support should involve proper staffing, easy access to resources, ongoing education, ergonomic supports, reporting systems, and leadership which fosters trust, accountability, and shared ownership of safety (72). Combined, workplace safety lays the structural and cultural groundwork for perioperative performance. Operational risk control, infection-prevention vigilance and organizational safety culture are all factors that influence the ability of operating room nurses to work with the precision, attention, and confidence necessary to provide high-risk surgical care (73).

## 5 Psychosocial stress, communication, and emotional labor

Psychosocial stress in operating room nursing arises from clinical urgency, professional accountability, team hierarchy, emotional demands, and the unpredictability of surgical work (74). The operating room differs from general wards because nursing performance must remain precise in a time-sensitive, highly interdependent environment where errors may have immediate consequences (75). This places psychosocial stress as a key factor in the wellbeing of the nurse and in the performance of the nurse in the perioperative environment (76). Stress in the operating room is not just created by the number of tasks that are to be performed. Still, it is also influenced by the communication requirements, the relationships and role expectations, and the constant pressure to be composed (27).

### 5.1 Psychosocial stressors in the operating room

The organization and pace of surgical care expose operating room nurses to substantial psychosocial stress. Nurses work under time pressure and high professional accountability while responding to unexpected procedural changes and the possibility of error (77). Instruments may be required without warning, a patient's condition may deteriorate, or communication within the team may become fragmented (78). Nurses must respond to these changes while protecting sterility, operating equipment accurately, and maintaining clear communication (79).

This situation is exacerbated by the psychological stresses of the operating room, where the nurse is likely to be involved in lengthy and stressful procedures with different groups of people, and varying expectations of interpersonal relationships (27). Recent evidence also has found a correlation between the perceived stress, anxiety and depression levels of operating room nurses with work experience and other contextual factors, implying that psychological strain is not only an individual phenomenon but also a reflection of the demanding work conditions (80). RNs with less experience may be at a higher risk, as they are still building up familiarity with the procedures, confidence, and understanding of working in a team. This underscores the need for mentorship, role clarity and psychological support in workforce development in the perioperative period (81). There is also a direct impact of psychosocial stress on performance. During a stressful time, there may be decreased attentional control, decreased working memory, increased emotional reactivity, and decreased communication during critical moments (82). In the operating room where anticipatory, vigilant and coordinated action is essential to good performance, unmanaged stress has the potential to impact both nurse wellbeing and patient-safety margins. So, psychosocial stress should not be considered merely a personal psychological problem, but as a system-level performance problem (83).

### 5.2 Communication, hierarchy, and relational strain

Communication is one of the most important pathways by which psychosocial stress can impact operating room nurses (74). The operating room is a multi-professional setting in which the nurse is required to communicate constantly with surgeons, anesthesiologists, technicians and others involved in the perioperative process. These exchanges are made under pressure and often involve quick decisions and incomplete information, competing priorities, or professional hierarchies (84). Effective communication is clear, respectful, and structured, which allows nurses to coordinate well, anticipate what may be required in the procedure and raise safety concerns (85). Stressed organizational communication leads to higher stress levels and possibly impaired safety performance if the communication is fragmented, hierarchical or dismissive (86).

Hierarchy plays a special role in operating room culture. Nurse–surgeon power dynamics, concerns about negative responses and punishment for mistakes can diminish nurses' tendency to raise concerns (87). This can cause a strain on the relationship and can help to prevent the timely exchange of safety information. The most recent meta-synthesis of literature on teamwork among operating room nurses and other healthcare providers revealed that the factors that foster the development of teamwork include role awareness, professional skills, leadership, psychological safety, patient-centered culture, and availability of adequate resources, whereas the barriers include hierarchy, individualistic behavior, punitive management, insufficient resources, and environmental constraints (88). These findings indicate that communication issues do not just reflect interpersonal difficulties but are influenced by the social and organizational structure of the operating room (89).

Communication can be further hindered by noise and multitasking. In a prospective cohort study, evidence revealed that communication failures happen in operating room settings where individuals are conducting complex cognitive activities, and that surgical phase, checklist-related communication, surgical count communication, multitasking, and the use of facemasks may impact communication failure (90). These failures are significant for operating room nurses because they can impair situational awareness, slow response times, add to redundant communication, and place an added cognitive burden. Thus, it is important to include communication quality as an integral part of psychosocial safety and clinical performance (91).

### 5.3 Emotional labor in perioperative nursing

Emotional labor is an important and under recognized aspect of operating room nursing (92). While the operating room is a technical environment, nurses working here need to constantly manage their emotions as well as urgency, hierarchy, uncertainty, patients' vulnerability, and team tension (93). They should behave calmly in emergency situations, act professionally when there is conflict, hide any signs of distress and be reassuring through communication and behavior. This emotional regulation is needed to keep teams stable, but when the emotional demands are great or are ongoing, and are not supported, this can lead to a psychological burden (94).

Emotional labor is frequently a hidden part of perioperative nursing (95). While continuing to perform technical tasks accurately, nurses can control fear of making mistakes, frustration over communication failures, anxiety during the unpredictable aspects of the operation, and emotional reaction when patients are harmed, or surgery is complicated (31). Nurses may exhibit “surface acting,” which is an outward calm demeanor despite inner turmoil when the culture and hierarchy of the group in which they work or the expectations of professional calm do not allow expression or emotion (96). This can over time lead to emotional exhaustion, diminished quality of work life, and burnout. The studies examining the strategies used for emotional labor by operating room nurses suggest a connection between emotional labor and quality of work life, underscoring its relevance to occupational wellbeing and sustainable performance (94, 97).

Emotional labor also is related to patient safety. Emotionally drained or relationally unfulfilled nurses may also have diminished abilities to assert themselves, maintain focus, and confidently participate in team communication (98). In contrast, psychologically safe workplaces that acknowledge emotional needs can foster more genuine conversations, improved coping mechanisms, and increased levels of professional engagement (99). Therefore, emotional labor must not be viewed as individual emotional burden; it must be included in the dialogue about work design, leadership, team culture and nurse wellbeing in perioperative systems (97).

## 6 Wellbeing, burnout, compassion fatigue, and quality of work life

Operating room nurses' wellbeing should be viewed as professional capacity and not just personal comfort. Nurses' psychological and physical state is a direct determinant of their capacity to pay attention, articulate clearly and to maintain composure and make timely decisions in the high-risk perioperative environment (100). Wellbeing is important in the operating room because it can be broken down by stresses, technical errors and a lack of coordination; therefore, compromised wellbeing can compromise the individual's performance and the reliability of the team (101). In this context, it is important to understand that burnout, compassion fatigue, occupational fatigue, and poor quality of work life are not only psychological outcomes but are markers of strain in the perioperative work system (26).

### 6.1 Burnout and compassion fatigue

Burnout and compassion fatigue pose substantial risks to the wellbeing and long-term retention of operating room nurses. Burnout is often linked to feelings of tiredness, lack of effectiveness, depersonalization and fatigue relating to work (102). Burnout in the operating room may be due to heavy workload, long operating hours, inadequate staffing, high expectations for outcomes, strain in communication with team members, and insufficient time to recuperate (103). Because perioperative work is safety-critical, burnout may affect not only nurses' wellbeing but also attention, communication, teamwork, and patient-safety behavior (104).

Compassion fatigue is very relevant in operating room nursing, where nurses go through repeated emotionally demanding patient-care situations, emergency surgery, unstable clinical situations and repeated invasive procedures (105). While the operating room can be considered a technological environment, the nurse is still vulnerable to patient fragility, surgical unpredictability, worst-case scenarios and the effects of high stakes care (106). High-level compassion fatigue is a current concern for operating room nurses and emotional strain can be considered as a measurable and clinically relevant workforce issue, emphasizing the importance of understanding and acting on emotional strain using machine learning and SHAP interpretation (107).

Workforce stability is also impacted by burnout and compassion fatigue. Emotionally exhausted nurses or nurses with chronic fatigue may report a decrease in job satisfaction, a decrease in level of professional engagement, and an increased intent to leave (108). This has a direct impact on perioperative systems as the current operating room nurse has specialized procedural knowledge, team familiarity and tacit safety expertise. Burnout also drives turnover, which leads to a loss of clinical memory, safety capacity and staffing levels (109). As such, burnout and compassion fatigue should be considered as organizational and patient-safety issues, rather than as purely psychological issues (110).

### 6.2 Quality of work life and workforce stability

Workforce stability depends on more than recruitment. It also requires retaining experienced nurses who can maintain team reliability, mentor colleagues, and preserve organizational safety culture (111). The factors identified as influencing perioperative nurse retention in recent qualitative evidence include organization support, workload, leadership and professional development opportunities (112). These findings indicate that a work environment that supports nurses' health and facilitates professional development and retention is needed for sustainable care in the perioperative setting (113). Thus, in modern surgical systems, quality of work life should be viewed as a strategic issue related to work and patients' safety (112).

## 7 Teamwork, hierarchy, and safety culture in the operating room

Teamwork is a principal mechanism through which operating room nurses contribute to safe and effective perioperative care. Surgical performance reflects the coordinated work of nurses, surgeons, anesthesia professionals, technicians, and support staff rather than the technical performance of one professional group (114). Within this team, operating room nurses maintain continuity by coordinating workflow, monitoring sterility, ensuring equipment readiness, and communicating patient-safety concerns. Their performance is therefore shaped by team relationships, communication norms, and the broader safety culture of the operating room (115).

### 7.1 Team-based nature of operating room performance

The performance of operating rooms should be thought of as a team process not an individual outcome. Surgery demands a synchronized approach, clarity of roles, trust between individuals and shared awareness of the situation (116). The nurse needs to be able to think ahead for the needs of surgery, communicate about instruments and equipment issues, maintain aseptic technique, adjust to changes in the procedure, and work with various professional groups as the surgeries unfold. The tasks cannot be optimally completed if communication is disjointed or roles are not clear (117, 118).

In the operating room, where the safety of the patient relies on a team's understanding of what is happening, who is doing what, and what the risks are, shared situational awareness is especially crucial (119). A shared mental model among nurses, surgeons and anesthetists allows the team to sense, understand and respond to threats, changes and opportunities for preventable harm (118). However, a lack of coordination can exacerbate delays, duplicate communications, mental strain, and risk of missing early indicators (120). Preoperative preparation and nursing teams dedicated to high-volume, high-tech surgical procedures like robotic-assisted surgery have demonstrated a positive impact on the efficiency of the operating room (18). This has helped to reinforce the notion that team organization is not just an administrative issue, but actually a factor that influences how well a team will perform clinically (121, 122).

### 7.2 Facilitators and barriers to effective teamwork

The operating room team functions effectively through professional respect of team members, role awareness, team familiarity, leadership, adequate resources and a patient-centered culture (123). These facilitators establish and maintain space for nurses to coordinate with confidence, to communicate clearly and to participate meaningfully in decision-making (23). The awareness of roles is particularly significant as the work in the operating room is interdependent between professional groups, tasks change rapidly, and responsibilities overlap (124). Effective communication between team members is more likely to happen when they have a clear grasp of their respective roles, and tasks are more likely to be completed on time and to the desired standard (42, 125).

Several factors were found to facilitate collaboration among operating room nurses and other practitioners, such as professional skills, awareness of roles, team familiarity, leadership, patient-centered culture, psychological safety, and resources (88). The same meta-synthesis also revealed that lack of resources and environmental constraints, hierarchy, individualistic behavior, and punitive management were obstacles to teamwork (88). The results of this study suggest that teamwork issues cannot be seen as individual interpersonal issues. Instead, they are related to the structure and culture of the operating room that affect the way professionals interact, how risks are communicated and how decisions are made (126).

### 7.3 Psychological safety and speaking-up behavior

Psychological safety in the operating room is paramount to teamwork as it will dictate whether nurses can voice concerns, question decisions, report near misses, and interrupt unsafe practices without risk of blame or retaliation. Speaking up may be difficult in hierarchical surgical environments, particularly when nurses fear that their concerns will be dismissed, discredited, or interpreted as challenges to authority (127). This is crucial because operating room nurses are on the front lines and can recognize early signs of sterility or equipment issues or counts or positioning issues or workflow issues or patient deterioration issues. Patient-safety opportunities can be lost if they cannot communicate these concerns (128).

Speaking-up behavior is situated in the context of safety culture. A robust safety culture fosters positive communication, shared responsibility, reporting without punishment, and leadership response (129). Conversely, a weak safety culture can lead to a silencing of the system, discourage questions, and diminish the visibility of system issues. More recent studies on potential implementation strategies to enhance safety climate in the operating room highlight the importance of health professionals' and leaders' actions in influencing behavior and organizational factors pertaining to safety (130). This clearly indicates that psychological safety is not just a team-level attitude; it is a leadership and management responsibility (130). Speaking up is both a performance behavior and a safety behavior for operating room nurses. It allows them to halt unsafe processes, resolve uncertainty, avoid errors, and engage in patient-safety decisions. However, speaking up requires an environment in which nursing expertise is valued, and concerns are viewed as contributions to collective safety rather than challenges to hierarchy (131). Creating a sense of psychological safety consequently depends on consistent leadership, structured communication mechanisms, debriefing protocols, non-punitive reporting systems, and interprofessional respect (132).

## 8 Distractions, cognitive load, and intraoperative performance

Distractions and cognitive load are important but often underestimated influences on operating room nursing performance. Nurses must continuously monitor the surgical field, instruments, equipment, patient positioning, documentation, and team communication (133). These activities occur in a dynamic environment in which procedural needs can change rapidly. Interruptions and simultaneous requests can therefore disrupt situational awareness and coordinated performance rather than merely causing inconvenience (134).

Anticipation, memory, sequencing, and rapid prioritization are essential aspects of a cognitive process that is used by the operating room nurse. Scrub and circulating nurses must track instruments, maintain sterility, set up equipment, complete surgical counts, document care, and communicate with surgeons and anesthesia professionals (135). These activities must be completed in a constant state of cognitive readiness, as this can be compromised by time delays, omission, or misinterpretation, resulting in unreliable workflow and patient safety. The more things nurses are asked to do at once, particularly during key procedural moments and equipment changes, surgical counts, emergencies, or transitioning between procedural steps, there is an increase in cognitive load (136). Communication failure, task delay or loss of safety cues can be a risk if the cognitive load is too great for their attentional capacity (137).

There is up-to-date evidence that distraction is associated with the experience and performance of operating room nurses. The study by Bozdemir et al. investigated the association of QWL and distractions among operating room nurses. It revealed that distraction is an important aspect of the work environment in the operating room (13). This is quite important because distractions not only cause productivity to come to a halt, but may also result in increased workloads, decreased job satisfaction, and psychological stress (138). Having to wait for instruments, or to follow up with patients or do paperwork while constantly interrupted by others, nurses are cognitively distracted from their sterile vigilance. These demands over time may lead to decreased quality of work and increased fatigue (13).

It is especially vital in the operating room that communication-related distractions are addressed (139). Communication failures may occur when complex cognitive tasks are performed amid multitasking, noise, checklist exchanges, surgical counts, and phase-specific demands. These findings are obviously relevant to the operating room nurse, as the effectiveness of the nurse's performance relies on accurate, timely and unambiguous communication (140). Frequent communications, poor communication, background noise, and other simultaneous oral exchanges can lead to greater cognitive load and a loss of a common picture of the situation. Communication can break down at crucial times, such as during surgical counts, requests for instruments or during transfers in procedure, which can reduce patient-safety margins (141, 142).

Documentation is also a source of cognitive load. Digital documentation is now a part of a growing number of perioperative processes and, while a digital solution can improve traceability, accountability, and continuity of information, it can also create additional cognitive burdens if systems are not integrated into the surgical workflow (143). Perioperative nurses' experiences indicate that digital documentation systems influence the work environment, workflow, and professional practice (144). In the operating room, documentation must be accurate and timely. Still, the nurse also may be required to document as well as be involved in other clinical tasks, team communication and changes in procedure. Documentation processes, therefore, may be poorly designed and compete with the need for real-time patient-safety monitoring (7, 145).

Technological complexity and workflow disruptions are also potential sources of distractions. Today, the operating room is filled with robotic platforms, digital systems and intelligent technologies, imaging devices and specialized equipment (146). While these technologies can enhance precision and workflow, if not integrated effectively, they can also pose a cognitive burden in cases where training, usability, and role clarity are lacking (147). The human factors literature on surgical workflow interruptions highlights the importance of how the integration of workflow and system safety is impacted when external or vendor-related interruptions disrupt the flow of a surgical procedure. These disruptions can pose extra coordination challenges and increase the risk of task fragmentation for operating room nurses (148, 149).

## 9 Ergonomics, robotic surgery, and physical strain

**Figure 3:**
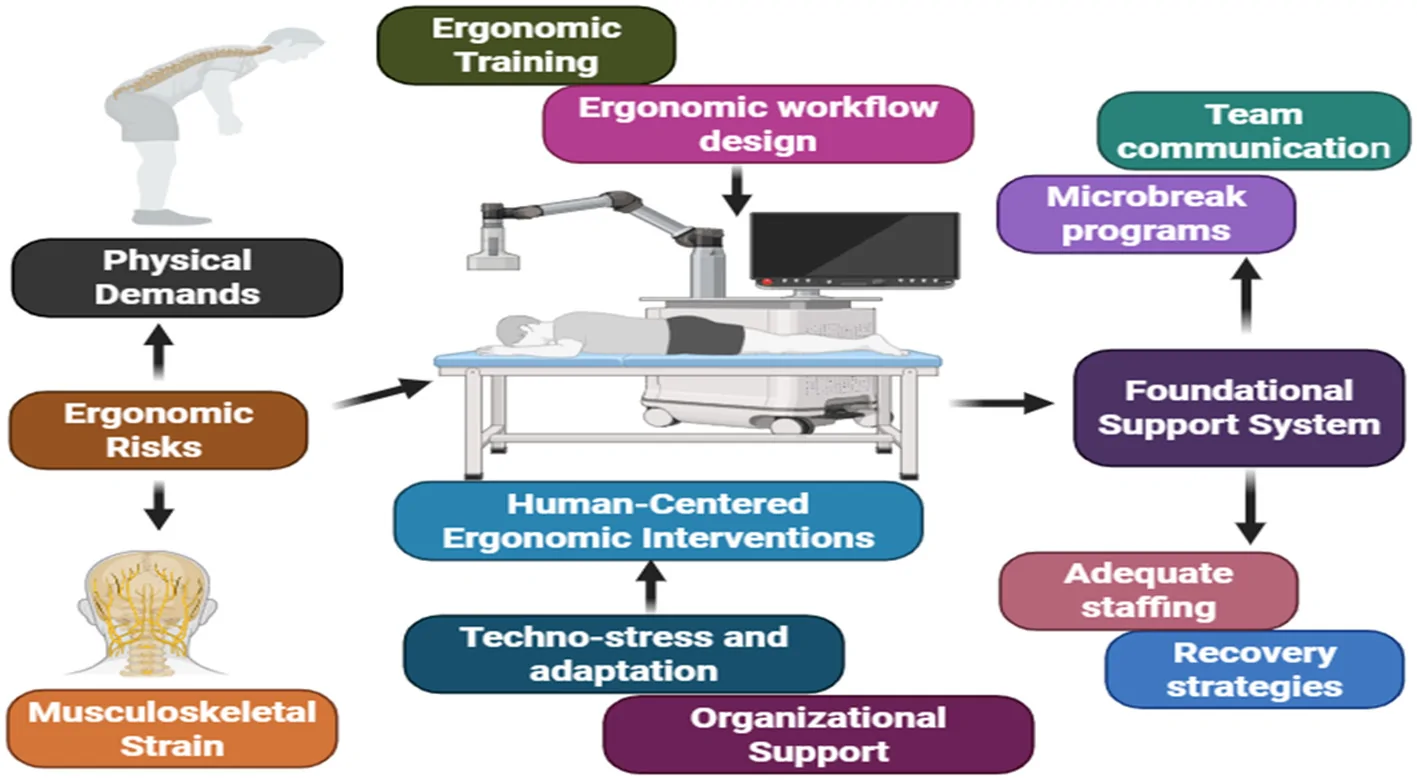
Ergonomic risks and robotic-surgery demands influence the physical and cognitive workload of operating room nurses. Human-centered design, ergonomic training, and organizational support help reduce strain, improve wellbeing, and strengthen perioperative performance and patient safety.

Ergonomics is a focal aspect of the work environment in operating room nursing as the work of the operating room is physically demanding and requires constant focus and technical precision (150). Often, operating room nurses are expected to work long shifts in cramped areas, assume static or awkward postures, repetitively operate the instruments, move equipment, and coordinate tasks during lengthy, complex procedures (151). These physical requirements are not secondary to the clinical performance: they impact fatigue, comfort, attention span and the flow of safe procedures. So, ergonomic risk should not only be viewed in the context of occupational health concerns, but also as a determinant of performance in relation to perioperative safety (Figure 3) (152).

### 9.1 Ergonomic risks in conventional operating rooms

The traditional operating room creates various ergonomic risks for nurses such as prolonged standing, repetitive upper-limb movements, awkward trunk and neck positions, heavy equipment handling, restricted movement, and static positions for extended procedures (153). Scrub nurses can be stationary, pass instruments, maintain sterility and react to surgical needs (154). Circulating nurses are subject to frequent transfers between equipment, documentation systems, supply areas and the surgical field. These tasks involve physical endurance and spatial awareness, as procedures can be lengthy or the operating room layout congested (155).

Among the most significant of the consequences of poor ergonomic conditions is musculoskeletal burden. Frequent work in non-neutral positions or tasks which are physically demanding without recovery can lead to neck and lower back, shoulder, wrist and leg discomfort (156). This includes the working posture of the operating room nurse, which has recently been identified as an ergonomic hazard, and the duration of the body posture during surgery in the OR posed a medium to high risk and musculoskeletal disorders were frequently reported in the neck and lower back area. These findings reiterate the importance of considering physical strain as a structural, rather than an individual adaptation problem, in OR work (157).

But performance may be affected by physical discomfort as well. During complex surgical tasks, the pain, fatigue and postural strain can lead to a decrease in stamina, concentration and responsiveness (158). Nurses who are physically exhausted may not be able to maintain good alertness, clear vocalization and hand-eye co-ordination, and may fail to predict the requirements for implements and be unable to react appropriately to unforeseen situations. As a result, the ergonomic risk is tightly coupled to the overall safety–wellbeing–performance continuum of this review (40).

### 9.2 Robotic surgery and changing physical demands

The introduction of robots in surgery has created a new set of technologies, spatial configurations, workflows and team roles that are challenging how surgery is traditionally performed (159). In the operating room, robotic surgery can lessen the physical burden of some tasks, such as better organization of the operating room or fewer manual tasks. Robotic surgery may also introduce new ergonomic and cognitive demands. Nurses are responsible for equipment preparation and robot docking, while also managing instrument exchanges and cables. Limited space around the robotic platform and communication with a console-based surgeon may create additional coordination challenges (160).

However, recent evidence has pointed to a change in the way that robotic surgery impacts ergonomic risk. A multicenter survey analysis indicated that although robotic surgery may offer some ergonomic advantages over laparoscopy, operating room nurses continue to experience musculoskeletal discomfort and gaps in ergonomic training (161). This means that surgical technology does not necessarily make things safer for workers. If not adequately equipped, trained, and aware of staff positioning, robotic surgery can change the physical requirements in another area of the nursing process (17).

Technostress and adaptation demands are other issues associated with robotic surgery. In a qualitative study of robotic surgery nurses, nurses who work in high-tech environments may have technology-related stressful experiences related to education, skill acquisition, group dynamics, work stress, and adaptation to future robotic practices. Ergonomic strain and technological stress may interact because complex equipment can increase both physical positioning demands and cognitive workload. Safe robotic practice for OR nurses thus not only involves technical competence, but also ergonomic awareness, team coordination and organizational support (162).

### 9.3 Ergonomic training and human-centered workflow design

A human-centered approach to operating room work requires systematic improvements in ergonomic design. The ergonomic safety of nurses should not be solely reliant on the nurse adjusting their own position or being able to cope with discomfort. Rather, health care establishments should plan operating rooms, equipment, job task distributions, and other health care procedures to minimize unnecessary physical stress. This includes the layout of equipment, space around the surgical area, tables and instrument stands that are adjustable, safe position of the patient, and minimization of unnecessary reaching and twisting of the body and the opportunity to rotate tasks when possible (40).

Education—ergonomic education is also essential. Nurses should be educated about body posture, body mechanics, safe handling of instruments, positioning of equipment, and early signs of musculoskeletal strain. It is found that ergonomics training can decrease risk-related behaviors and increase awareness of musculoskeletal risks among operating room nurses. Training in robotic surgery should not be limited to the operation of the device, but also to understanding safe positioning when docking, the exchange of instruments, cable management, emergency conversion, and communication with the console surgeon and bedside team (157).

Human-centered Workflow Design also requires Leadership commitment. Nurse managers and perioperative leaders should monitor musculoskeletal concerns, seek nurses' feedback on operating room layouts, and incorporate microbreaks or recovery strategies during prolonged procedures. Structured microbreak interventions have been recently evaluated in operating room nurses and could play a role in musculoskeletal health, psychological wellbeing, and patient-safety-related outcomes. This shows that ergonomic protection does not have to stand alone from performance enhancement; it is integral to the design of surgical systems that allow for sustainable human performance (163).

In this way, a mature approach to ergonomics is associated with physical safety, technological adaptation and reliability of performance. Both conventional and robotic operating rooms require systems that protect nurses' physical health while enabling sustained alertness, coordination, and clinical accuracy. With the advancement of surgical technology, the ergonomic design should still focus on the nurses who maintain the continuity of the operation and supervise patient safety (164).

## 10 Competency, empowerment, technology readiness, and workforce sustainability

Competency, empowerment, technology readiness, and workforce sustainability are central to operating room nurses' performance in contemporary healthcare settings. Professional performance in the operating room extends beyond mastery of procedural skills and includes clinical judgment, communication, emotional regulation, adaptability, and safe technology use. The modern and technological operating room and the perioperative care delivered by operating room nurses (ORNs) require extra specialized training and empowerment as well as resilience, confidence and professional organizational support to ensure provision of high-quality care (165). Evidence-informed strategies to address these challenges and strengthen operating room nursing practice are summarized in Table 2.

**Table 2:** Evidence-informed strategies to strengthen safety, wellbeing, and performance among operating room nurses.

Problem area	Recommended strategy	Implementation level	Expected outcome	References
Limited perioperative competency	Structured OR-specific training, competency checklists, simulation-based learning, and procedure-specific education	Education/unit level	Improved technical competence, confidence, procedural readiness, and patient-safety performance	(184, 185)
Infection-prevention gaps	Continuous education on aseptic technique, sterile field management, instrument processing, and evidence-based guidelines	Clinical/team level	Stronger infection-prevention behavior, improved vigilance, and reduced risk of intraoperative contamination	(186)
Low confidence among novice OR nurses	Mentorship, supervised practice, peer support, and progressive responsibility models	Unit/management level	Improved professional confidence, reduced transition stress, and safer progression to independent practice	(187)
Weak empowerment and speaking-up culture	Leadership support, psychological safety training, non-punitive reporting, and interprofessional respect	Organizational/team level	Greater nurse participation in safety decisions, earlier risk communication, and stronger team reliability	(188, 189)
Burnout and reduced resilience	Workload monitoring, recovery opportunities, resilience-building support, counseling access, and fatigue management	Management/organizational level	Lower emotional exhaustion, improved wellbeing, and stronger workforce stability	(190)
Technology-readiness gaps	AI, robotic surgery, and digital documentation training with hands-on practice and usability feedback	System/unit level	Safer technology adoption, reduced digital stress, and improved workflow integration	(86)
Robotic surgery adaptation challenges	Dedicated robotic nursing teams, role clarification, docking protocols, troubleshooting training, and ergonomic planning	OR/team level	Improved robotic workflow efficiency, fewer disruptions, and stronger team coordination	(171)
Retention risk	Career pathways, recognition of OR expertise, continuing education, leadership development, and staffing support	Policy/organizational level	Improved retention, stronger institutional knowledge, and sustainable perioperative workforce capacity	(191)
Fragmented professional development	Integration of competency, wellbeing, technology readiness, and retention strategies into one workforce plan	Health-system level	More resilient, competent, and future-ready OR nursing workforce	(192)

### 10.1 Perioperative competency and professional development

Perioperative competency is multidimensional. It includes technical skill, procedural knowledge, infection-prevention practice, accurate documentation, and effective communication. It also requires nurses to anticipate procedural needs and respond appropriately when unexpected events occur. These responsibilities demand specialized perioperative education rather than general nursing preparation alone (22).

Perioperative practice presents a number of unique risks, technologies, workflows and team structures, so specialized operating room training is particularly important. Specialized operating room training, clinical experience, empowerment, resilience, and work self-efficacy were found to be predictors of operating room nurses' perioperative competency (166). This indicates that competency is not a result of mere time in practice, but also from structured learning, psychological resources and support from professionals. Likewise, the evidence on infection prevention highlights the need for professional competence, evidence-based guidance, resources, and supportive teamwork as prerequisites for safe and secure intraoperative work (167). Thus, simulation-based education, continuing professional development and procedure-specific training are key components of enhancing the performance of the operating room. Aseptic technique, instrument handling, surgical counts, emergency preparedness, robotic and digital technologies, communication protocols and human factors should all be included. Competency development should also be continued as a process, as there are quick changes in surgical techniques, technologies and patient-safety expectations. Developing a mature perioperative workforce involves education pathways that foster novice nurses, update experienced nurses and prepare teams for new technologies.

### 10.2 Empowerment, resilience, and self-efficacy

Empowerment is crucial to operating room nurses' participation in safety-related decision-making. Nurses with empowerment are more likely to speak out about concerns, ask questions about unsafe practices, adhere to guidelines, and participate in interprofessional coordination. Conversely, nurses who lack autonomy or professional recognition may hesitate to voice concerns even when they recognize a potential risk. Empowerment thus is tightly coupled to patient safety, psychological safety and professional performance in high-stakes surgical contexts (168).

Operating room nurses' response to stress, uncertainty, technological complexity and demanding procedures is also influenced by resilience and self-efficacy. Resilience helps to cope with stress, and self-efficacy enhances confidence in executing complex procedures and communicating with other health care providers. Resilience and work self-efficacy were determined as predictors of perioperative competency; Fu et al. suggested that psychological resources are part of professional competency. This implies that competency is not only of the technical nature but also has confidence, emotion control, independence and pressure handling (169).

Organizational conditions are necessary to support empowerment. Leadership, mentoring, feedback, and ongoing education and a non-punitive safety culture can be used to increase nurses' confidence and participation in safety processes. This support is especially important for junior operating room nurses, who may find it difficult to communicate with senior staff or respond to unfamiliar situations. Structured supervision and mentorship can help build professional identity, reduce anxiety, and provide a safe space to transition to independent practice (170).

### 10.3 Technology readiness, retention, and sustainable workforce planning

In the age of robotic surgery, artificial intelligence, digital documentation, complex equipment platforms, automated monitoring and advanced imaging, technology readiness is becoming more and more important in the operating room. These technologies can enhance workflow, documentation, decision support and improve perioperative safety; at the same time, they also present new training requirements and cognitive demands. While AI and precision nursing hold promise for revolutionizing the field of perioperative safety and surgical outcomes, the ethical, human-centric, and workflow-sensitive integration of AI systems is crucial for realizing these benefits (148).

One of the other technology readiness aspects is robotic surgery. OR nurses should understand how to set up the robotic equipment, docking, instrument changes, troubleshooting, emergency conversion and communicating with the surgeon at the console. The data collected from robotic surgery indicates that structured preparation of the nurses and their competence in the management of the robotic system can help improve workflow efficiency with the use of dedicated teams for the perioperative period. However, the use of robotic and digital technologies cannot be viewed as standalone technological solutions, but needs to be complemented by training, usability evaluation, role clarification, ergonomic planning and inter-professional coordination (159, 171).

The final component is workforce sustainability. Competency and technology readiness cannot be maintained without a stable and adequately supported workforce. Experienced operating room nurses possess procedural knowledge, team familiarity, safety awareness, and mentoring capacity. When experienced nurses leave, organizations lose institutional knowledge, procedural familiarity, mentorship capacity, and workflow reliability (172). The workload, support, leadership and professional development opportunities were identified as factors impacting nurse retention in the perioperative environment. This highlights the need for workforce planning that integrates wellbeing, career development, mentorship, organizational support, and recognition of perioperative expertise. A sustainable workforce planning approach should, therefore, be joined with professional development, psychological support, technology readiness, and retention planning. For high-performing OR, it is essential to have clinically competent nurses, feel empowered to be part of the safety discussion, are prepared for technology change and are supported to remain in the OR (173).

## 11 Integrated synthesis, limitations, and future directions

This review consolidates workplace safety, wellbeing and performance of operating room nurses as three aspects of perioperative care. Today's operating rooms demand that nurses possess technical accuracy, infection control awareness, communication skills, emotional stability, stamina and ergonomics, and technological awareness to manage high-stress surgical situations. Their performance is therefore influenced by their individual competence, staffing, leadership, teamwork, safety culture, workload and organizational support (115).

At the clinical-practice level, operating room teams should strengthen structured communication, infection-prevention vigilance, role clarity, and the management of distractions during critical procedural phases. At the management level, priorities include adequate staffing, balanced working hours, mentorship, continuing education, and psychological support. Health-system policies should also address ergonomic protection, fatigue management, responsible technology implementation, artificial-intelligence governance, and nurse retention (174).

This review has several limitations. First, its thematic narrative design did not employ the systematic literature-screening, study-selection, and risk-of-bias assessment procedures typically used in systematic reviews. Consequently, the selection, organization, and interpretation of the evidence may have been influenced by reviewer judgment and may not represent all relevant publications. Second, the included literature was heterogeneous in terms of study design, clinical setting, participant characteristics, and outcome measures, which limited direct comparisons and precluded statistical pooling. Finally, much of the available evidence was based on cross-sectional, qualitative, or descriptive studies, limiting causal inference and the generalizability of the conclusions across different perioperative settings and healthcare systems.

Future research should move beyond descriptive and cross-sectional designs by using longitudinal and intervention-based approaches. Increased evidence is required regarding the impact of OR nurse safety and wellbeing on patient outcomes, such as surgical site infections, intraoperative mistakes, delays, adverse events, and postoperative complications. Future research should also create OR-specific measurements of cognitive load, emotional labor, ergonomic strain, technology readiness and safety performance. Additional research is needed on the nurse's side to explore the possibilities of robot surgery, AI nursing, psychological safety measures, preventing compassion fatigue, and retaining nurses.

## 12 Conclusion

Operating room nurses have a pivotal role in contemporary surgical care. Their contribution to patient safety depends on technical and aseptic competence, effective communication, physical and emotional capacity, and reliable teamwork. This review demonstrates that workplace safety, nurse wellbeing, and professional performance are not separate concerns. They are interrelated system-level priorities that influence the quality, reliability, and sustainability of perioperative care.

Improving outcomes requires more than individual resilience or isolated educational interventions. Healthcare organizations must reduce physical hazards, psychological strain, cognitive overload, ergonomic risk, and problems associated with poorly integrated technologies. Leadership should simultaneously support adequate staffing, psychological safety, continuing professional development, infection-prevention competence, responsible technology adoption, and workforce retention. Treating operating room nurse safety and wellbeing as patient-safety priorities can strengthen teamwork, clinical reliability, and the sustainability of the perioperative workforce.
